# Genetic Characterization of *Caiman crocodilus* (Crocodilia: Alligatoridae) on Gorgona Island, Colombia

**DOI:** 10.3390/biology14091227

**Published:** 2025-09-09

**Authors:** Natalia Londoño, Raúl Ernesto Sedano-Cruz, Alan Giraldo

**Affiliations:** Grupo de Investigación en Ecología Animal, Departamento de Biología, Facultad de Ciencias Naturales y Exactas, Universidad del Valle, Cali 760032, Colombia; brenda.n.londono@correounivalle.edu.co (N.L.); alan.giraldo@correounivalle.edu.co (A.G.)

**Keywords:** spectacled caiman, genetic variation, haplogroups, phylogenetic, island conservation

## Abstract

This study examines the genetic variation and structure of the spectacled caiman (*Caiman crocodilus*) on Gorgona Island, Colombia, compared to continental populations. We analyzed 178 partial Cytochrome b gene sequences, most of which were obtained from GenBank, and identified 23 haplogroups, with five of these specifically found on the Island. Phylogenetic analysis using maximum likelihood placed *C. crocodilus*, including the Gorgona Island population, in a distinct monophyletic group. Genetic structure analysis identified two main clusters, with Gorgona Island caimans primarily assigned to the Trans-Andean cluster. The haplogroup network illustrates the two major groups, with a maximum of 12 mutational steps between them. Additionally, Tajima’s D statistic suggests an excess of rare alleles in the spectacled caiman. Genetic differentiation across regions suggests historical isolation, likely shaped by geographical barriers and limited gene flow. The distinct genetic patterns of island populations highlight their disparity in terms of evolutionary dynamics and conservation importance. Further genomic analysis is recommended to explore demographic history. Conservation strategies should prioritize the maintenance of genetic diversity to mitigate the effects of isolation, while also incorporating insights from the species’ biogeographic history. Our findings highlight the unique contribution of the small population in Gorgona Island to the species’ spatial genetic structure.

## Simple Summary

This study examines the mithocondrial genetic diversity of the spectacled caiman (*Caiman crocodilus*) on Gorgona Island, Colombia, and provides a preliminary phylogeographic assessment. By analyzing partial sequences of the Cytochrome *b* gene, our research compares the genetic structure of the island’s caiman population with that of the mainland. Our findings reveal significant genetic overlap between Gorgona caimans and those from northern Central America to Colombia, suggesting multiple colonization events. We identified 23 haplogroups across the species’ vast distribution, with five of these specifically found on the Island, highlighting a pronounced genetic separation between the Trans- and Cis-Andean regions. These results underscore the importance of Gorgona Island as a significant ecological context, emphasizing the broader implications for *C. crocodilus* conservation and biogeography. Our study calls for further research into the demographic history and selective pressures affecting these populations in order to enhance conservation efforts.

## 1. Introduction

The phylogeographic patterns observed across widely geographically distributed reptiles can provide invaluable insights into their biogeographical history, particularly concerning the westernmost side of the Andean region. These data are especially significant within biodiversity conservation zones, such as the Chocó biogeographic region. Spanning from eastern Panama to northern Peru, and encompassing the Colombian Pacific coast, the Chocó region is a global hotspot for species richness, serving as a vital natural corridor for population connectivity [1]. It is one of the planet’s wettest areas and is known for its high endemism, hosting impressive plant and vertebrate diversity [1]. Within this rich biodiversity, reptiles—particularly crocodilians—occupy an essential ecological niche as apex predators and key global indicators of freshwater ecosystems [2].

Among these, the spectacled caiman (*Caiman crocodilus*) stands out as an ecologically significant species, with both high cultural value and potential as a flagship species for conservation [3]. The spatial genetic structure of the spectacled caiman—especially that of the population on Gorgona Island, Colombia—is of particular interest. Situated in the eastern Pacific Ocean off the southwestern Colombian coast, this island is renowned for its unique biodiversity; its ecological significance has led to its designation as a national park [4,5,6].

Gorgona Island has historical records of *C. crocodilus* presence dating back to the mid-20th century [7]. However, these records are sparse, consisting mainly of occasional sightings and counts of individuals in aquatic habitats, of a relatively small population (unpulished) [8]. Given the limited data, studying the genetic diversity of this population is critical to understanding its lineage evolutionary history. Our study carries broader implications beyond Gorgona Island, offering insights into caiman colonization processes in a continental island environment. This not only contributes to our understanding of *C. crocodilus* genetics but also enhances our knowledge of the species’ regional biogeography. There is limited available information on *C. crocodilus* populations in island environments (e.g., [9,10,11]), and this study seeks to fill this gap.

Our primary aim is to examine a small set of genetic data for *C. crocodilus* individuals from Gorgona Island, focusing on the Cytochrome *b* gene, a commonly used molecular marker for analyzing the spectacle caiman genetic structure [12,13]. By examining the genetic variation among the Gorgona Island caimans and comparing them with mainland populations, we aim to uncover preliminary phylogeographic evidence of the history of these caimans on a continental island. This study also evaluates how local data align with previously proposed phylogenies, providing insights into the spatial distribution of genetic variation and its broader implications for local and regional conservation efforts.

## 2. Materials and Methods

Gorgona Island (2°9′ N and 78°2′ W) is a continental island located in the southern region of the Colombian Pacific. The nearest distance to the continental coast is 35 km, and its dimensions are 8.5 km in length, 2.5 km in width, and a maximum elevation of 338 m above sea level. Gorgona boasts a freshwater system with 25 permanent streams where *C. crocodilus* resides and reproduces [8]. Single caudal crest scales deposited in the herpetological collection UV-C 81 of Universidad del Valle were used to carry out this research; these were obtained while marking individuals captured at night in different streams as part of a population-monitoring study. Furthermore, we also included samples provided by the Alexander von Humboldt Biological Resources Research Institute, Colombia (IAvH) from the Magdalena River basin, a Colombian mainland environment providing additional *de novo* sequences. Genomic DNA extraction was performed using the Thermo Scientific^®^ kit (Waltham, MA, USA). For the amplification of the mitochondrial genetic marker Cytochrome *b* (Cyt *b*) through Polymerase Chain Reaction (PCR), a final volume of 25 μL was prepared, containing the following: 2.5 μL Tris Buffer HCL, 1.25 μL MgCl_2_, 1.25 μL of each Primer, 2.5 μL DNTP, 15.05 μL dd H_2_O, 1 μL DNA template, and 0.2 μL Taq. The following primers used were: L14211 (5′AAG ATC TGA ARA ACC YCG TTG 3′) [12], and CB3H (5′GGC AAA TAG GAA RTA TCA 3′) [14]. Amplification was carried out with a thermocycler, starting with an initial denaturation at 94 °C (2 min), followed by shorter denaturation at 94 °C (45 s), annealing at 53 °C (45 s), extension at 72 °C (1 min), and final extension at 72 °C (1.5 min) by 35 cycles. Amplified products were separated by molecular weight through electrophoresis using a 2% agarose gel at 130 volts for 50 min and visualized under a transilluminator. Finally, the products were sent to Macrogen Inc. (Gangnam-gu, Republic of Korea) for purification and sequencing using the BigDye reaction (Applied Biosystems TM, Foster City, CA, USA).

A consensus sequence was obtained for each pair obtained from both primers using the Sequencher 4.1 program [15]. These sequences were compared with reference sequences from the gene bank using BLAST v2.12 [16] in order to verify their identity. Subsequently, the consensus sequences were submitted to the Genbank genetic database [17]. A database was constructed combining the genetic information available in Genbank with the new records representing genetic information for Gorgona Island and the Magdalena River basin in Colombia (Table S1). Finally, we performed an alignment of all compiled sequences for the study, and haplotype richness was calculated based on a nucleotide identity criterion using the Usearch v11 program [18].

To determine haplotype richness, we applied a 0.997% nucleotide identity criterion across an alignment of 685 base pairs of Cytochrome *b* (Cyt *b*) sequences. We identified 23 haplogroups, a number that differs from previous *Caiman crocodilus* Cyt *b* studies (e.g., Vasconcelos et al. reported 38 [19], Venegas-Amata et al. reported 31 [12], and Balaguera et al. reported 66 [20]). This disparity in haplogroup counts hinders direct comparison because previous studies often omit their specific grouping criteria. By clearly stating our nucleotide identity threshold, we provide essential methodological detail for future comparative research. Sequence alignments were analyzed separately for each study group to determine the dN/dS ratio and enable subsequent comparisons. Analyses were performed using R statistical software version 4.3.1 and the Biological Sequences Retrieval and Analysis package [21].

The genetic structure of *C. crocodilus* was studied at a continental scale using Arlequin v3.5 [22] and further using the geographical information associated with each sequence in the alignment. To investigate biogeographical patterns, a haplogroup network was constructed using a parsimony criterion with the ‘Median-Joining’ method in the PopArt program [23]. Additionally, genetic structure analysis was implemented to determine the assignment of Gorgona individuals to population clusters. For this purpose, the Geneland program version 4.9.2 was employed within the statistical software R version 4.3.1. The Geneland algorithm utilizes both genetic information and the geographical location of each sample [24]. This model assumes that the spatial domain of each population in terms of genetic variability can be approximated by initially joining a few polygonal subdomains. The model corresponds to a spatial structure pattern, which can be anticipated when assuming that differentiation is influenced by gene flow restriction or by a limitation in dispersal potential due to the presence of physical barriers [24]. The analysis was conducted with k = 4 as the maximum number of populations, and the inference was independently repeated ten times.

We deduced the phylogenetic relationships among all individuals via maximum likelihood (ML) using *Alligator mississipiensis*, *Paleosuchus trigonatus*, *Melanosuchus niger*, *Caiman latirostris* and *Caiman yacare* as outgroups. The ML tree topology was estimated with IQ-Tree v.2.0 [25]. We selected the best substitution model using the IQ-Tree feature ModelFinder. Node support was calculated using 10,000 UltraFast Bootstrap pseudoreplicates. Values > 95% were considered good.

## 3. Results

### 3.1. Genetic Variation

**Figure 1:**
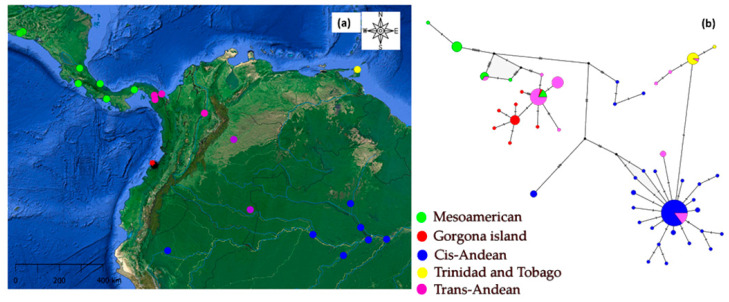
(**a**) Locations where partial mtDNA sequences were obtained for *Caiman crocodilus* (from our study and GenBank) Green: Mesoamerica, Red: Gorgona, Blue-Magenta: Cis-Andean (Perú, Brazil, Colombia: Palmarito), Yellow: Trinidad and Tobago, and Magenta: Trans-Andean (Colombia: Chocó-Magdalena). (**b**) Haplogroup network based on partial Cyt *b* gene sequences. In the network, each color corresponds to a DNA sequence found at a specific location on the map, and circle sizes are proportional to the sample size.

Fifteen sequences were obtained from Gorgona Island, and three were obtained from the Rio Magdalena basin in Colombia. Each sequence had a length ranging from 518 to 685 base pairs. Searching in GenBank allowed us to complement the Cyt *b* dataset with 36 sequences from individuals in other localities in Colombia, and 127 sequences from individuals distributed across Mexico, El Salvador, Costa Rica, Panama, French Guiana, Peru, Trinidad and Tobago, and Brazil (Table S1, Figure 1a) [12,19].

A total of 178 accessions from Cyt *b* sequences were aligned on a 685 base pair fragment of the Cyt *b* gene. The alignment presents less than 25% missing data. Additionally, 65.7% of the loci were invariable sites, while 10% (68 bp) corresponded to informative loci. From all accessions in the alignment, we identified 23 haplogroups using a similarity criterion of ≥99% between the partial Cyt *b* sequences, as determined by using Usearch v11 [18]. Seventeen haplogroups were identified in the Trans-Andean region, with five of these specifically found on Gorgona Island. The significance of the estimated values for Tajima’s D statistic [26] in each locality (Table 1) suggest that the Cyt *b* marker meets the neutrality condition for the Trans- and Cis-Andean groups. The negative values observed in most of the groupings suggest a potential excess of rare alleles.

The haplotype network (Figure 1b) shows two major clusters of haplogroups that correspond to the Trans- and Cis-Andean clades, separated by the Andean ranges, both of which are supported by phylogenetic analysis. The greatest number of mutational steps separating major clusters is 12, as observed in several cases. Firstly, this 12-step distance is found between haplotype 12—identified as a sequence found in Costa Rica—the Mesoamerican region, and sequences from *Cis-Andes* in Brazil.

Also, a 12-step mutational distance is evident among haplogroups in the *cis*-Andean region, specifically between haplotypes 16 and 22, found in Brazil and Peru, respectively, and haplotype 6 from Panama. Finally, haplotype 23, found on Gorgona Island, also exhibits a genetic distance of 12 mutational steps from haplotype 3 taken from a continental Colombian population in the Cis-Andean locality of Palmarito, Casanare.

### 3.2. Genetic Structure

**Figure 2:**
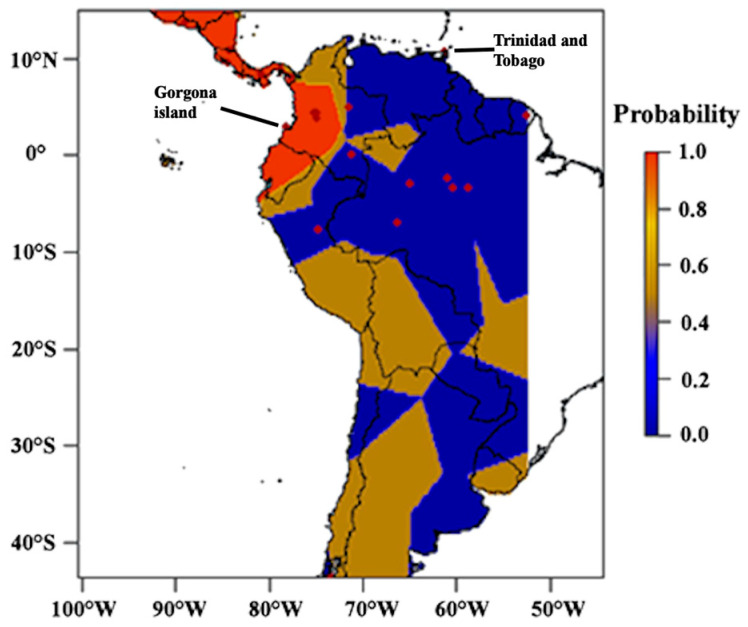
Spatial assignment of *Caiman crocodilus* mtDNA partial sequences based on posterior probabilities from Geneland analysis. The map illustrates the degree of individual assignment to the Pacific Domain biogeographic region (Trans-Andean), as defined by Morrone [27]. Darker shades of orange represent higher probabilities of assignment to this region.

The individual assignment suggests that the most likely number of genetic clusters within *C. crocodilus* is k = 5 populations (Figure 2). This analysis, implemented in Geneland [24], reveals a pronounced pattern of genetic variation, distinctly separating the Trans- and Cis-Andean regions. This partition accounts for up to 35.5% of the spatial genetic variation distribution (AMOVA, Fst = 0.3475, *p* < 0.05). Sequences from caimans on Gorgona Island are assigned to the cluster primarily composed of *Trans*-Andean sequences.

**Figure 3:**
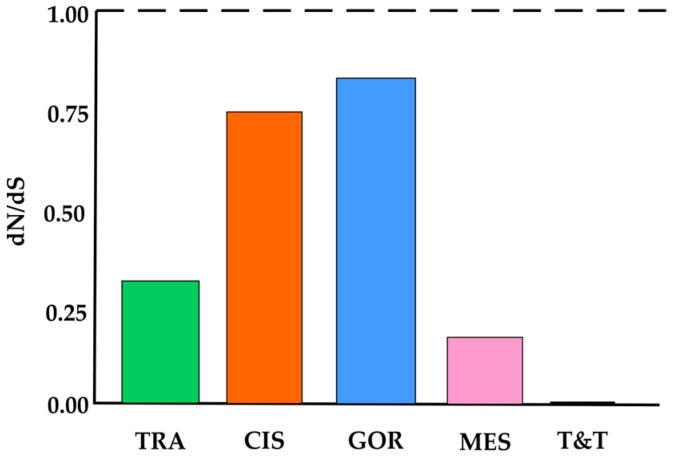
Mean ratio of non-synonymous to synonymous substitutions (dN/dS) in the mitochondrial Cyt *b* gene fragment across five populations. Dashed line: value expected under neutrality. TRA: Trans-Andean, CIS: Cis-Andean, GOR: Gorgona island, MES: Mesoamerican, T&T: Trinidad and Tobago.

The average dN/dS ratio of the mitochondrial Cyt *b* gene was estimated for each grouping. All groups exhibited values below 1, consistent with evolution under purifying selection. However, the Gorgona Island (0.83) and Cis-Andean (0.75) groupings showed the highest ratios, approaching values expected under neutral evolution, while the Trinidad and Tobago Island as a group showed a lack of non-synonymous substitutions in the analyzed gene fragment (dN/dS = 0) (Figure 3).

### 3.3. Phylogenetic Inference

**Figure 4:**
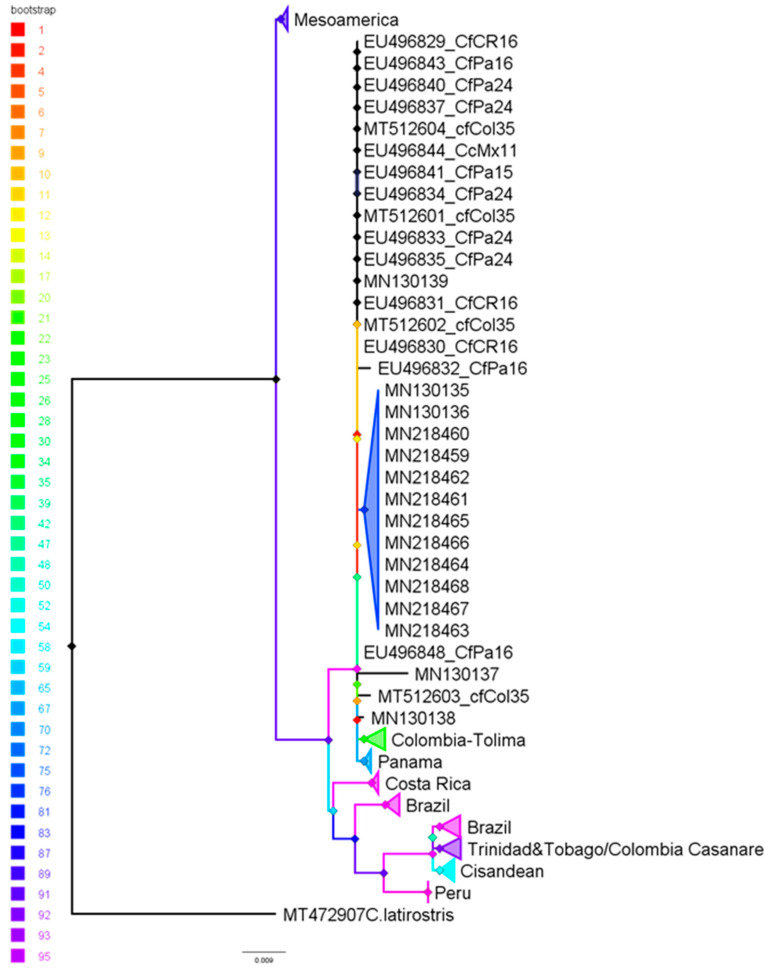
Gene tree reconstruction showing the relationships among partial Cyt *b* sequences from *Caiman crocodilus* individuals sampled across their distribution, from Mexico to Brazil, including the study population from Gorgona Island. The method employed here is maximum likelihood (ML), with bootstrap support for groupings indicated by coloration gradient as per the figure’s scale.

The most suitable nucleotide substitution model for the Cyt *b* gene was GTR + F + I. In this context, the phylogenetic reconstruction places *C. crocodilus* as a well-supported clade, with *C. latirostris* serving as the outgroup. The genealogy reveals an initial divergence between haplogroups from Mesoamerica, specifically Mexico and El Salvador. The remaining sequences in the analysis form a second divergent event: a sister clade to MES consisting of sequences from Costa Rica, Panama, and Colombia, including Gorgona Island. Additionally, individuals from the inter-Andean region of Colombia, specifically the Tolima region, are part of this second clade, which also contains other Trans-Andean sequences from Colombia, Gorgona Island, and Panama. In contrast, sequences from Colombia’s Casanare and Río Apaporis regions are more closely related to the Cis-Andean cluster, which is primarily composed of sequences from Brazil but also includes some from Trinidad and Tobago, islands located in the southern Caribbean and part of the West Indies (Figure 4).

## 4. Discussion

Our sequences from Colombia further complement existing genetic knowledge for this reptile in the Trans-Andean region [12,13,19,20,27,28,29,30], offering valuable insights into the extensive biogeographic history of this species. The genetic diversity and spatial structure of *C. crocodilus cyt b* gene align with findings from several previous studies [28,29,30]. However, the number of reported haplogroups in our study (23) differs from findings of previous studies using the Cyt *b* marker for *C. crocodilus*, such as Vasconcelos et al. [19], who identified 38 haplotypes, and Venegas-Anaya et al. [12], who proposed 31. The most significant contrast arises when comparing our results with those of Balaguera et al. [20], who identified 66 haplotypes. The challenge in comparing these estimates stems from the absence of reported information regarding the criteria employed by other authors to group sequences in their studies. Nonetheless, the reduction in sequence variation within haplogroups offers certain technical advantages, including mitigating errors associated with aligning a large number of sequences and minimizing redundancy effects during subsequent nucleotide variation analyses [18]. This approach has been previously explored in other taxa, where it successfully estimated haplogroups within alignments containing over 1600 partial mtDNA sequences [31].

In the Trans-Andean region, we identified 17 out of 23 total haplogroups, revealing a remarkable disparity in genetic diversity compared to the Cis-Andean range. Notably, five of these haplogroups were found in Gorgona Island, emphasizing its importance as a reservoir for caiman haplotypic variation in the eastern tropical Pacific coast. Whether this represents a pattern of endemism requires further, more extensive sampling along the Pacific coast of Colombia, Ecuador, and Peru. It is striking that this 23 km^2^ island, estimated to harbor fewer than 83 caimans [8], exhibits such high level of genetic diversity within the Trans-Andean range for this species. We predict that if only a few distinct haplotypes characterize the broader Pacific coast, this would strongly suggest a potential for ongoing differentiation processes on Gorgona Island, likely driven by the isolation and reduced population sizes typical of insular environments. Further analysis of whole genomic data to investigate the demographic history of these populations could provide deeper insights into the observed genetic patterns as a primary hypothesis. Moreover, our individual assignment analysis revealed two major groups, consistent with the geographical clades identified in previous studies. We found that the Trans-Andean population (where Gorgona Island is located), can be associated with the geographical area of the Pacific Domain [32]. This zoogeographic region coincides with an area of endemism for mammals [33].

Our study reinforces the Andes Mountains as a major biogeographic barrier for *C. crocodilus* [12], evidenced by a 12-mutational step difference between Trans-Andean (Costa Rican) and Cis-Andean (Colombian/Brazilian) haplogroups. This significant genetic differentiation, consistent with previous findings, points to low maternal dispersal or restricted gene flow. Strikingly, Gorgona Island’s haplotype 23 also showed a 12-mutational step distance from a Cis-Andean continental population Palmarito, Casanare, Colombia. This suggests a genetic distinctiveness across the Trans-Andean range and warrants further investigation into a more localized insular caiman colonization. Island populations often exhibit lower genetic diversity than their mainland counterparts, a pattern largely driven by restricted gene flow and geographic isolation [34,35,36].

Despite anecdotal sightings of *Caiman crocodilus* far off Colombia’s Pacific coast, suggesting greater saltwater tolerance, the species is still thought to have limited ability to cross marine barriers [19]. This reinforces isolation by distance or through strong female site fidelity. Consequently, island populations typically have reduced effective population sizes and, as a result, lower and more homogenized genetic variability [29].

Studies on *C. crocodilus* in Trinidad and Tobago have shown reduced genetic diversity, likely due to historical separation from mainland populations across marine gaps [29]. Such conditions can also lead to the fixation of rare or uncommon haplotypes in island populations and may or may not indicate long-term divergence, based on what remains following local extinction events. These complex phenomena are sometimes difficult to discern based only on maternal inherited markers such as mtDNA. Nonetheless, this type of process may be occurring on Gorgona Island. Despite the island harboring a substantial subset of regional haplotype diversity, insularity and limited effective dispersal events with coastal *C. crocodilus* populations could be shaping distinct genetic patterns in the island population. However, carrying out a further, more detailed comparison using whole-genome sequence data of coastal populations in Colombia, Ecuador and Peru would help clarify this pattern.

## 5. Conclusions

Building on previous research, our study provides new insights into the broad genetic range of maternal-inherited mtDNA genes within *C. crocodilus* populations. We reveal the genetic diversity specific to Gorgona Island, including five haplogroups potentially found along the Colombian and Ecuadorian coasts. This study provides sufficient information to confirm that the caiman haplotypes present on Gorgona Island are likely the result of multiple colonization events, leading to the accumulation of haplotypes of *C. crocodilus* that recently diverged in the Trans-Andean region.

The Trans-Andean region harbors 73.9% of the species’ genetic variation in the Cyt *b* gene, emphasizing the need for further research on the factors driving genetic diversity and population structure across this area. The low genetic diversity observed in island populations reflects outcomes that are typical of insularized *C. crocodilus* populations, likely shaped by long-term isolation and reduced gene flow. To preserve the evolutionary potential and option value that these isolated populations represent—particularly in the face of global warming—conservation strategies must prioritize maintaining genetic diversity and, where feasible, facilitating gene flow. Future studies examining demographic dynamics and the ecological pressures acting on island populations will be critical for designing data-driven, long-term conservation strategies for sustaining endemic haplotypes and preventing further genetic erosion. Although our phylogenetic reconstruction was based on maximum likelihood methods, future analyses incorporating whole genomic datasets will benefit of implementing Bayesian inference to further validate clade relationships and assess their posterior probabilities.

## Figures and Tables

**Table 1:** Genetic variation in *Caiman crocodilus* in the studied regions. N: sample size, H: haplotype groups, Hd: haplotypic diversity, π: nucleotidic diversity and standard deviation, Tajima’s D statistic [26], *p*: neutrality test *p*-value.

Region	N	H	Hd	π	Tajima’s D	*p*
Trans-Andean	34	17	0.7219	0.012189 ± 0.006434	0.32835	0.70700
Gorgona	15	8	0.7195	0.003751 ± 0.002525	−2.021300	0.01200
Trinity and Tobago	12	2	0.1667	0.000730 ± 0.000750	−1.62929	0.04600
Cis-Andean	118	32	0.7333	0.011278 ± 0.005861	−0.90917	0.19000

## Data Availability

The raw data supporting the conclusions of this article will be made available by the authors on request.

## Supplementary Materials

The following supporting information can be downloaded at: https://www.mdpi.com/article/10.3390/biology14091227/s1, Table S1: Molecular sequences used in this Study and retrieved from GenBank, including geographic locations.

## References

[1] Myers N., Mittermeier R., Mittermeier C., da Fonseca G.A., Kent J. (2000). Biodiversity hotspots for conservation priorities. Nature.

[2] Martin S., Balian E.V., Lévêque C., Segers H., Martens K. (2007). Global diversity of crocodiles (Crocodilia, Reptilia) in freshwater. Freshwater Animal Diversity Assessment. Developments in Hydrobiology.

[3] Bartlett R., Bartlett P. (2003). Reptiles and Amphibians of the Amazon.

[4] Díaz J.M., Pinzón J.H., Perdomo A.M., Barrios L.M., López-Victoria M., Barrios L.M., López-Victoria M. (2001). Generalidades. Gorgona Marina: Contribución al Conocimiento de una Isla Única.

[5] Giraldo A., Valencia B. (2012). Isla Gorgona: Paraíso de Biodiversidad y Ciencia.

[6] Giraldo A., Diazgranados M.C., Gutiérez-Landázuri C.F. (2014). Isla Gorgona, enclave estratégico para los esfuerzos de conservación en el Pacífico Oriental Tropical. Rev. Biol. Trop..

[7] Medem F., von Prahl H., Guhl F., Grögl M. (1979). Los anfibios y reptiles de las islas Gorgona y Gorgonilla. Gorgona.

[8] Londoño B.N. (2023). Atributos Poblacionales de *Caiman crocodilus* en un Ambiente Insular del Pacífico Colombiano. Master’s Thesis.

[9] Balaguera-Reina S., González-Maya J. (2009). Estructura poblacional, abundancia y uso del hábitat de *Caiman crocodilus fuscus* (Cope, 1868) en la Vía Parque Isla Salamanca, Caribe Colombiano. Rev. Biol. Mar. Oceanogr..

[10] Forero-Medina G., Castaño-Mora O.V., Rodriguez-Melo M. (2006). Ecología de *Caiman crocodilus fuscus* en San Andrés Isla, Colombia: Un estudio preliminar. Caldasia.

[11] Mohammed R.S. (2015). Distribution of the Caiman, *Caiman crocodilus* on Tobago. Living World J. Trinidad Tobago Field Nat. Club.

[12] Venegas-Anaya M., Crawford A., Escobedo-Galván A., Sanjur O., Densmore L., Bermingham E. (2008). Mitochondrial DNA phylogeography of *Caiman crocodilus* in Mesoamerica and South America. J. Exp. Zool. Part A Ecol. Genet. Physiol..

[13] Amavet P.S., Pacheco-Sierra G., Uhart M.M., Prado W.S., Siroski P.A. (2023). Phylogeographical analysis and phylogenetic inference based on the cytochrome *b* gene in the genus *Caiman* (Crocodylia: Alligatoridae) in Central and South America. Biol. J. Linn. Soc..

[14] Palumbi S.R., Hillis D.M., Moritz C., Mable B.K. (1996). Nucleic acids II: The polymerase chain reaction. Molecular Systematics.

[15] Arbor A. (2010). Sequencher.

[16] Johnson M., Zaretskaya I., Raytselis Y., Merezhuk Y., McGinnis S., Madden T.L. (2008). NCBI BLAST: A better web interface. Nucleic Acids Res..

[17] Benson D.A., Cavanaugh M., Clark K., Karsch-Mizrachi I., Lipman D.J., Ostell J., Sayers E.W. (2013). GenBank. Nucleic Acids Res..

[18] Edgar R. (2010). Search and clustering orders of magnitude faster than BLAST. Bioinformatics.

[19] Vasconcelos W.R., Hrbek T., Da Silveira R., de Thoisy B., Marioni B., Farias I.P. (2006). Population genetic analysis of *Caiman crocodilus* (Linnaeus, 1758) from South America. Genet. Mol. Biol..

[20] Balaguera-Reina S., Vargas-Ramírez M., Ordóñez-Garza N., Hernández-González F., Densmore L.D. (2020). Unveiling the mystery: Assessing the evolutionary trajectory of the Apaporis caiman population (*Caiman crocodilus apaporiensis*, Medem 1955) via mitochondrial molecular markers. Biol. J. Linn. Soc..

[21] Charif D., Lobry J.R., Bastolla U., Porto M., Roman H.E., Vendruscolo M. (2007). SeqinR 1.0-2: A Contributed Package to the R Project for Statistical Computing Devoted to Biological Sequences Retrieval and Analysis. Structural Approaches to Sequence Evolution.

[22] Excoffier L., Lischer H.E.L. (2010). Arlequin Suite Ver 3.5: A new series of programs to perform population genetics analyses under Linux and Windows. Mol. Ecol. Resour..

[23] Leigh J.W., Bryant D. (2015). PopART: Full-feature software for haplotype network construction. Methods Ecol. Evol..

[24] Guillot G., Mortier F., Estoup A. (2005). Geneland: A computer package for landscape genetics. Mol. Ecol..

[25] Minh B.Q., Schmidt H.A., Chernomor O., Schrempf D., Woodhams M.D., von Haeseler A., Lanfear R. (2020). IQ-TREE 2: New models and efficient methods for phylogenetic inference in the genomic era. Mol. Biol. Evol..

[26] Tajima F. (1989). Statistical method for testing the neutral mutation hypothesis by DNA polymorphism. Genetics.

[27] Dessauer H.C., Glenn T.C., Densmore L.D. (2002). Studies on the molecular evolution of the Crocodylia: Footprints in the sands of time. J. Exp. Zool..

[28] Roberto I.J., Bittencourt P.S., Muniz F.L., Hernández-Rangel S.M., Nóbrega Y.C., Ávila R.W., Souza B.C., Álvarez G., Miranda-Chumacero G., Campo Z. (2020). Unexpected but unsurprising lineage diversity within the most widespread Neotropical crocodilian genus *Caiman* (Crocodylia, Alligatoridae). Syst. Biodivers..

[29] Balaguera-Reina S.A., Konvalina J.D., Mohammed R.S., Gross B., Vazquez R., Moncada J.F., Ali S., Hoffman E.A., Densmore L.D. (2021). From the river to the ocean: Mitochondrial DNA analyses provide evidence of spectacled caimans (*Caiman crocodilus* Linnaeus 1758) mainland–insular dispersal. Biol. J. Linn. Soc..

[30] Jiménez-Alonso G., Balaguera-Reina S.A., Hoyos M., Bloor P. (2023). Phylogenetic and phylogeographic insights on Trans-Andean spectacled caiman populations in Colombia. Mar. Freshw. Res..

[31] Gil-Vargas D.L., Sedano-Cruz R.E. (2019). Genetic variation of avian malaria in the tropical Andes: A relationship with the spatial distribution of hosts. Malar. J..

[32] Morrone J. (2014). Cladistic biogeography of the Neotropical region: Identifying the main events in the diversification of the terrestrial biota. Cladistics.

[33] Noguera-Urbano E.A., Escalante T. (2015). Áreas de endemismo de los mamíferos (Mammalia) neotropicales. Acta Biol. Colomb..

[34] Meyer N.F., Balkenhol N., Dutta T., Hofman M., Meyer J.Y., Ritchie E.G., Alley C., Beranek C., Bugir C.K., Callen A. (2021). Beyond species counts for assessing, valuing, and conserving biodiversity: Response to Wallach et al. 2019. Conserv. Biol..

[35] Holmes I.A., Mautz W.J., Davis Rabosky A.R. (2016). Historical environment is reflected in modern population genetics and biogeography of an island endemic lizard (*Xantusia riversiana reticulata*). PLoS ONE.

[36] Trumbo D.R., Funk W.C., Pauly G.B., Robertson J.M. (2021). Conservation genetics of an island-endemic lizard: Low Ne and the critical role of intermediate temperatures for genetic connectivity. Conserv. Genet..

