# Supplementary material for: Genetic Characterization of Caiman crocodilus (Crocodilia: Alligatoridae) on Gorgona Island, Colombia
Source: Biology (Basel). 2025 Sep 9;14(9):1227. doi: 10.3390/biology14091227 (PMC12467642; doi:10.3390/biology14091227)
Supplement: Supplementary file 1 [file biology-14-01227-s001.zip › biology-3633601-supplementary.pdf]

Table S1. Molecular sequences used in this Study and retrieved from GenBank. Including Geographic Origins.

GenBank	Alignment Code	Country	Locality	Geographic Coordinates	Reference
AY462456.1	AY462456_CBr44	Brazil	Purus River	4°43′ S & 62°21′ W	*C. crocodilus*
AY462457.1	AY462457_CBr44	Brazil	Purus River	4°43′ S & 62°21′ W	*C. crocodilus*
AY462458.1	AY462458_CBr44	Brazil	Purus River	4°43′ S & 62°21′ W	*C. crocodilus*
AY462459.1	AY462459_CBr44	Brazil	Purus River	4°43′ S & 62°21′ W	*C. crocodilus*
AY462460.1	AY462460_CBr44	Brazil	Purus River	4°43′ S & 62°21′ W	*C. crocodilus*
AY462461.1	AY462461_CBr44	Brazil	Purus River	4°43′ S & 62°21′ W	*C. crocodilus*
AY462462.1	AY462462_CBr44	Brazil	Purus River	4°43′ S & 62°21′ W	*C. crocodilus*
AY462463.1	AY462463_CBr44	Brazil	Purus River	4°43′ S & 62°21′ W	*C. crocodilus*
AY462464.1	AY462464_CBr44	Brazil	Purus River	4°43′ S & 62°21′ W	*C. crocodilus*
AY462465.1	AY462465_CBr44	Brazil	Purus River	4°43′ S & 62°21′ W	*C. crocodilus*
AY462466.1	AY462466_CBr44	Brazil	Purus River	4°43′ S & 62°21′ W	*C. crocodilus*
AY462467.1	AY462467_CBr44	Brazil	Purus River	4°43′ S & 62°21′ W	*C. crocodilus*
AY462468.1	AY462468_CBr41	Brazil	Purus River	4°43′ S & 62°21′ W	*C. crocodilus*
DQ246626.1	DQ246626_CBr44	Brazil	Purus River	4°43′ S & 62°21′ W	*C. crocodilus*
DQ246627.1	DQ246627_CBr44	Brazil	Purus River	4°43′ S & 62°21′ W	*C. crocodilus*
DQ246628.1	DQ246628_CBr44	Brazil	Purus River	4°43′ S & 62°21′ W	*C. crocodilus*
AY462469.1	AY462469_CBr41	Brazil	Janauacá Lake	3°25'29" S & 60°16'54" W	*C. crocodilus*
AY462470.1	AY462470_CBr41	Brazil	Janauacá Lake	3°25'29" S & 60°16'54" W	*C. crocodilus*
AY462471.1	AY462471_CBr41	Brazil	Janauacá Lake	3°25'29" S & 60°16'54" W	*C. crocodilus*
AY462472.1	AY462472_CBr41	Brazil	Janauacá Lake	3°25'29" S & 60°16'54" W	*C. crocodilus*
AY462473.1	AY462473_CBr41	Brazil	Janauacá Lake	3°25'29" S & 60°16'54" W	*C. crocodilus*
AY462474.1	AY462474_CBr41	Brazil	Janauacá Lake	3°25'29" S & 60°16'54" W	*C. crocodilus*
AY462475.1	AY462475_CBr41	Brazil	Janauacá Lake	3°25'29" S & 60°16'54" W	*C. crocodilus*
AY462476.1	AY462476_CBr41	Brazil	Janauacá Lake	3°25'29" S & 60°16'54" W	*C. crocodilus*
AY462477.1	AY462477_CBr41	Brazil	Janauacá Lake	3°25'29" S & 60°16'54" W	*C. crocodilus*
AY462478.1	AY462478_CBr41	Brazil	Janauacá Lake	3°25'29" S & 60°16'54" W	*C. crocodilus*
AY462479.1	AY462479_CBr41	Brazil	Janauacá Lake	3°25'29" S & 60°16'54" W	*C. crocodilus*
DQ246629.1	DQ246629_CBr41	Brazil	Janauacá Lake	3°26′ S & 60°17′ W	*C. crocodilus*
DQ246630.1	DQ246630_CBr41	Brazil	Janauacá Lake	3°26′ S & 60°17′ W	*C. crocodilus*
DQ246631.1	DQ246631_CBr41	Brazil	Janauacá Lake	3°26′ S & 60°17′ W	*C. crocodilus*
DQ246637.1	DQ246637_CAA39	Brazil	Anavilhanas	2°23′41″ S & 60°55′14″ W	*C. crocodilus*
DQ246639.1	DQ246639_CAA39	Brazil	Anavilhanas	2°23′41″ S & 60°55′14″ W	*C. crocodilus*
DQ246640.1	DQ246640_CAA39	Brazil	Anavilhanas	2°23′41″ S & 60°55′14″ W	*C. crocodilus*
DQ246641.1	DQ246641_CAA39	Brazil	Anavilhanas	2°23′41″ S & 60°55′14″ W	*C. crocodilus*
DQ246642.1	DQ246642_CAA39	Brazil	Anavilhanas	2°23′41″ S & 60°55′14″ W	*C. crocodilus*
DQ246643.1	DQ246643_CAA39	Brazil	Mamirauá Lake	2°59′ S & 64°53′ W	*C. crocodilus*
DQ246644.1	DQ246644_CBr36	Brazil	Mamirauá Lake	2°59′ S & 64°53′ W	*C. crocodilus*
DQ246645.1	DQ246645_CBr36	Brazil	Mamirauá Lake	2°59′ S & 64°53′ W	*C. crocodilus*
DQ246646.1	DQ246646_CBr36	Brazil	Mamirauá Lake	2°59′ S & 64°53′ W	*C. crocodilus*
DQ246647.1	DQ246647_CBr36	Brazil	Mamirauá Lake	2°59′ S & 64°53′ W	*C. crocodilus*
DQ246648.1	DQ246648_CBr36	Brazil	Mamirauá Lake	2°59′ S & 64°53′ W	*C. crocodilus*
DQ246649.1	DQ246649_CBr36	Brazil	Mamirauá Lake	2°59′ S & 64°53′ W	*C. crocodilus*
DQ246650.1	DQ246650_CBr36	Brazil	Mamirauá Lake	2°59′ S & 64°53′ W	*C. crocodilus*
DQ246651.1	DQ246651_CBr38	Brazil	Uacá River	3°45′ N & 51°36′ W	*C. crocodilus*
DQ246652.1	DQ246652_CBr38	Brazil	Uacá River	3°45′ N & 51°36′ W	*C. crocodilus*
DQ246653.1	DQ246653_CBr38	Brazil	Uacá River	3°45′ N & 51°36′ W	*C. crocodilus*
DQ246654.1	DQ246654_CBr38	Brazil	Uacá River	3°45′ N & 51°36′ W	*C. crocodilus*
DQ246655.1	DQ246655_CBr38	Brazil	Uacá River	3°45′ N & 51°36′ W	*C. crocodilus*
DQ246658.1	DQ246658_CBr41	Brazil	Tapará	1°35'58.98" S & 52°4'13.22" W	*C. crocodilus*
DQ246659.1	DQ246659_CBr41	Brazil	Tapará	1°35'58.98" S & 52°4'13.22" W	*C. crocodilus*

DQ246660.1	DQ246660_CBr41	Brazil	Tapará	1°35'58.98" S & 52°4'13.22" W	*C. crocodilus*
EU161660.1	EU161660_CBr43	Brazil	Madeira River	3°22′32″ S & 58°46′23″ W	*C. crocodilus*
EU161661.1	EU161661_CBr43	Brazil	Madeira River	3°22′32″ S & 58°46′23″ W	*C. crocodilus*
EU161662.1	EU161662_CBr43	Brazil	Madeira River	3°22′32″ S & 58°46′23″ W	*C. crocodilus*
EU161663.1	EU161663_CBr43	Brazil	Madeira River	3°22′32″ S & 58°46′23″ W	*C. crocodilus*
EU161664.1	EU161664_CBr43	Brazil	Madeira River	3°22′32″ S & 58°46′23″ W	*C. crocodilus*
EU161665.1	EU161665_CBr43	Brazil	Madeira River	3°22′32″ S & 58°46′23″ W	*C. crocodilus*
EU161666.1	EU161666_CBr43	Brazil	Madeira River	3°22′32″ S & 58°46′23″ W	*C. crocodilus*
EU161667.1	EU161667_CBr43	Brazil	Madeira River	3°22′32″ S & 58°46′23″ W	*C. crocodilus*
EU161668.1	EU161668_CBr43	Brazil	Madeira River	3°22′32″ S & 58°46′23″ W	*C. crocodilus*
EU161669.1	EU161669_CBr43	Brazil	Madeira River	3°22′32″ S & 58°46′23″ W	*C. crocodilus*
EU161670.1	EU161670_CBr43	Brazil	Madeira River	3°22′32″ S & 58°46′23″ W	*C. crocodilus*
EU161671.1	EU161671_CBr43	Brazil	Madeira River	3°22′32″ S & 58°46′23″ W	*C. crocodilus*
AY462480.1	AY462480_CGF38	French Guiana	Approuague River	4°40′ N & 52°10′ W	*C. crocodilus*
AY462481.1	AY462481_CGF38	French Guiana	Approuague River	4°40′ N & 52°10′ W	*C. crocodilus*
AY462482.1	AY462482_CGF38	French Guiana	Approuague River	4°40′ N & 52°10′ W	*C. crocodilus*
AY462483.1	AY462483_CGF38	French Guiana	Approuague River	4°40′ N & 52°10′ W	*C. crocodilus*
AY462484.1	AY462484_CGF38	French Guiana	Approuague River	4°40′ N & 52°10′ W	*C. crocodilus*
AY462485.1	AY462485_CGF38	French Guiana	Approuague River	4°40′ N & 52°10′ W	*C. crocodilus*
AY462486.1	AY462486_CGF38	French Guiana	Approuague River	4°40′ N & 52°10′ W	*C. crocodilus*
AY462487.1	AY462487_CGF38	French Guiana	Approuague River	4°40′ N & 52°10′ W	*C. crocodilus*
DQ246633.1	DQ246633_CGF38	French Guiana	Approuague River	4°40′ N & 52°10′ W	*C. crocodilus*
DQ246634.1	DQ246634_CGF38	French Guiana	Approuague River	4°40′ N & 52°10′ W	*C. crocodilus*
DQ246635.1	DQ246635_CGF38	French Guiana	Approuague River	4°40′ N & 52°10′ W	*C. crocodilus*
DQ246636.1	DQ246636_CGF38	French Guiana	Approuague River	4°40′ N & 52°10′ W	*C. crocodilus*
DQ246656.1	DQ246656_CPe41	Peru	Pacaya-Samíria	4°19′ S & 76°55′ W	*C. crocodilus*
DQ246657.1	DQ246657_CPe41	Peru	Pacaya-Samíria	4°19′ S & 76°55′ W	*C. crocodilus*
EU496856.1	EU496856_CfPe46	Peru	Rio Ucayali	4°30'01" S & 73°26'03" W	*C. c. crocodilus*
EU496855.1	EU496855_CfPe46	Peru	Rio Ucayali	4°30'01" S & 73°26'03" W	*C. c. crocodilus*
EU496854.1	EU496854_CfPe46	Peru	Rio Ucayali	4°30'01" S & 73°26'03" W	*C. c. crocodilus*
EU496853.1	EU496853_CfPe46	Peru	Rio Ucayali	4°30'01" S & 73°26'03" W	*C. c. crocodilus*
EU496850.1	EU496850_CfPa16	Panama	San Juan River	8°16'51"N & 80°51'11" W	*C. c. fuscus*
EU496849.1	EU496849_CfPa16	Panama	San Juan River	8°16'51"N & 80°51'11" W	*C. c. fuscus*
EU496848.1	EU496848_CfPa16	Panama	Chagres River	9°05′ N & 79°41′ W	*C. c. fuscus*
EU496844.1	EU496844_CcPa11	Panama	Panama Chanel	12°20'00" N & 79°55'00" W	*C. c. fuscus*
EU496842.1	EU496842_CfPa15	Panama	San Juan River	8°16'51"N & 80°51'11" W	*C. c. fuscus*
EU496843.1	EU496843_CfPa16	Panama	Panama Chanel	9° 20' 00" N & 79° 55' 00" W	*C. c. fuscus*
EU496841.1	EU496841_CfPa15	Panama	La Maestra River	8° 51' 07" N &78° 47' 45" W	*C. c. fuscus*
EU496840.1	EU496840_CfPa24	Panama	Balsa River	8° 13' 00" N & 77° 58' 00" W	*C. c. fuscus*
EU496839.1	EU496839_CfPa16	Panama	San Juan River	8°16'51"N & 80°51'11" W	*C. c. fuscus*
EU496838.1	EU496838_CfPa16	Panama	San Juan River	8°16'51"N & 80°51'11" W	*C. c. fuscus*
EU496837.1	EU496837_CfPa24	Panama	Tuira River	8° 20' 42" N & 78° 03' 58" W	*C. c. fuscus*
EU496835.1	EU496835_CfPa24	Panama	Armila River	8° 40' 00" N & 77° 28' 00" W	*C. c. fuscus*
EU496834.1	EU496834_CfPa24	Panama	Armila River	8° 40' 00" N & 77° 28' 00" W	*C. c. fuscus*
EU496833.1	EU496833_CfPa24	Panama	Armila River	8° 40' 00" N & 77° 28' 00" W	*C. c. fuscus*
EU496817.1	EU496817_CfCR15	Costa Rica	San Juan River	10°48′02″ N & 84°12′39″ W	*C. c. fuscus*
EU496818.1	EU496818_CfCR15	Costa Rica	San Juan River	10°48′02″ N & 84°12′39″ W	*C. c. fuscus*
EU496819.1	EU496819_CfCR15	Costa Rica	San Juan River	10°48′02″ N & 84°12′39″ W	*C. c. fuscus*
EU496820.1	EU496820_CfCR15	Costa Rica	San Juan River	10°48′02″ N & 84°12′39″ W	*C. c. fuscus*
EU496821.1	EU496821_CfCR15	Costa Rica	San Juan River	10°48′02″ N & 84°12′39″ W	*C. c. fuscus*
EU496829.1	EU496829_CfCR16	Costa Rica	Parrita River	9°31′17″ N & 84°19′44″ W	*C. c. fuscus*
EU496830.1	EU496830_CfCR16	Costa Rica	Parrita River	9°31′17″ N & 84°19′44″ W	*C. c. fuscus*
EU496831.1	EU496831_CfCR16	Costa Rica	Parrita River	9°31′17″ N & 84°19′44″ W	*C. c. fuscus*
EU496832.1	EU496832_CfPa16	Costa Rica	Parrita River	9°31′17″ N & 84°19′44″ W	*C. c. fuscus*

EU496822.1	EU496822_CfES14	El Salvador	San Salvador	13°41′ N & 89°11′ W	*C. c. fuscus*
EU496823.1	EU496823_CfES14	El Salvador	San Salvador	13°41′ N & 89°11′ W	*C. c. fuscus*
EU496824.1	EU496824_CfES14	El Salvador	San Salvador	13°41′ N & 89°11′ W	*C. c. fuscus*
EU496825.1	EU496825_CfES14	El Salvador	San Salvador	13°41′ N & 89°11′ W	*C. c. fuscus*
EU496827.1	EU496827_CfES14	El Salvador	San Salvador	13°41′ N & 89°11′ W	*C. c. fuscus*
EU496828.1	EU496828_CfES14	El Salvador	San Salvador	13°41′ N & 89°11′ W	*C. c. fuscus*
EU496826.1	EU496826_CfES14	El Salvador	Ilobasco	13°49'59.88"N & 88°55'0.12" W	*C. c. fuscus*
EU496847.1	EU496847_CcMx11	Mexico	Tapachula	14°54′ N & 92°16′ W	*C. c. chiapasius*
EU496846.1	EU496846_CcMx11	Mexico	Tapachula	14°54′ N & 92°16′ W	*C. c. chiapasius*
EU496845.1	EU496845_CcMx11	Mexico	Tapachula	14°54′ N & 92°16′ W	*C. c. chiapasius*
MZ218125.1	MZ218125_CcTT27	Trinidad-Tobago		10°41'30.49" N & 61°13'37.59" W	*C. crocodilus*
MZ218126.1	MZ218126_CcTT27	Trinidad-Tobago		10°41'30.49" N & 61°13'37.59" W	*C. crocodilus*
MZ218127.1	MZ218127_CcTT27	Trinidad-Tobago		10°41'30.49" N & 61°13'37.59" W	*C. crocodilus*
MZ218128.1	MZ218128_CcTT27	Trinidad-Tobago		10°41'30.49" N & 61°13'37.59" W	*C. crocodilus*
MZ218129.1	MZ218129_CcTT27	Trinidad-Tobago		10°41'30.49" N & 61°13'37.59" W	*C. crocodilus*
MZ218130.1	MZ218130_CcTT27	Trinidad-Tobago		10°41'30.49" N & 61°13'37.59" W	*C. crocodilus*
MZ218131.1	MZ218131_CcTT27	Trinidad-Tobago		10°41'30.49" N & 61°13'37.59" W	*C. crocodilus*
MZ218132.1	MZ218132_CcTT27	Trinidad-Tobago		10°41'30.49" N & 61°13'37.59" W	*C. crocodilus*
MZ218133.1	MZ218133_CcTT27	Trinidad-Tobago		10°41'30.49" N & 61°13'37.59" W	*C. crocodilus*
MZ218134.1	MZ218134_CcTT27	Trinidad-Tobago		10°41'30.49" N & 61°13'37.59" W	*C. crocodilus*
MZ218135.1	MZ218135_CcTT27	Trinidad-Tobago		10°41'30.49" N & 61°13'37.59" W	*C. crocodilus*
MZ218136.1	MZ218136_CcTT27	Trinidad-Tobago		10°41'30.49" N & 61°13'37.59" W	*C. crocodilus*
MT316395.1	MT316395_CcCol36	Colombia	Apaporis River	0°4'23.4" N & 71°13'37.59" W	*C. crocodilus*
MT316396.1	MT316396_CcCol36	Colombia	Apaporis River	0°4'23.4" N & 71°13'37.59" W	*C. crocodilus*
MT316397.1	MT316397_CcCol36	Colombia	Apaporis River	0°4'23.4" N & 71°13'37.59" W	*C. crocodilus*
MT316398.1	MT316398_CcCol36	Colombia	Apaporis River	0°4'23.4" N & 71°13'37.59" W	*C. crocodilus*
MT316399.1	MT316399_CcCol36	Colombia	Apaporis River	0°4'23.4" N & 71°13'37.59" W	*C. crocodilus*
MT316400.1	MT316400_CcCol36	Colombia	Apaporis River	0°4'23.4" N & 71°13'37.59" W	*C. crocodilus*
MT316401.1	MT316401_CcCol36	Colombia	Apaporis River	0°4'23.4" N & 71°13'37.59" W	*C. crocodilus*
MT316402.1	MT316402_CcCol36	Colombia	Apaporis River	0°4'23.4" N & 71°13'37.59" W	*C. crocodilus*
MT512613.1	MT512613_CcCol29	Colombia	Wisirare, Casanare	4°53'30" N & 71°26'27" W	*C. c. crocodilus*
MT512594.1	MT512594_CcCol29	Colombia	Palmarito, Casanare	4°49'43" N & 71°39'56" W	*C. c. crocodilus*
MT512595.1	MT512595_CcCol29	Colombia	Palmarito, Casanare	4°49'43" N & 71°39'56" W	*C. c. crocodilus*
MT512614.1	MT512614_CcCol29	Colombia	Wisirare, Casanare	4°53'30" N & 71°26'27" W	*C. c. crocodilus*
MT51259.1	MT512596_CcCol29	Colombia	Palmarito, Casanare	4°49'43" N & 71°39'56" W	*C. c. crocodilus*
MT51259.1	MT512597_CcCol29	Colombia	Palmarito, Casanare	4°49'43" N & 71°39'56" W	*C. c. crocodilus*
MT51259.1	MT512598_CcCol29	Colombia	Palmarito, Casanare	4°49'43" N & 71°39'56" W	*C. c. crocodilus*
MT512599.1	MT512599_CcfCol35	Colombia	Prado, Tolima	4°42'49" N & 74°53'14" W	*C. c. fuscus*
MT512600.1	MT512600_CcfCol35	Colombia	Prado, Tolima	3°42'49" N & 74°53'14" W	*C. c. fuscus*
MT512601.1	MT512601_CcfCol35	Colombia	Prado, Tolima	3°42'49" N & 74°53'14" W	*C. c. fuscus*
MT512602.1	MT512602_CcfCol35	Colombia	Prado, Tolima	3°42'49" N & 74°53'14" W	*C. c. fuscus*
MT512603.1	MT512603_CcfCol35	Colombia	Prado, Tolima	3°42'49" N & 74°53'14" W	*C. c. fuscus*
MT512604.1	MT512604_CcfCol35	Colombia	Prado, Tolima	3°42'49" N & 74°53'14" W	*C. c. fuscus*
MT512587.1	MT512587_CcfCol35	Colombia	Ibague, Tolima	4°25'39" N & 75°08'03" W	*C. c. fuscus*
MT512606.1	MT512606_CcfCol35	Colombia	San Luis, Tolima	4°07'59" N & 75°05'20" W	*C. c. fuscus*
MT512588.1	MT512588_CcfCol35	Colombia	Ibague, Tolima	4°25'39" N & 75°08'03" W	*C. c. fuscus*
MT512607.1	MT512607_CcfCol35	Colombia	San Luis, Tolima	4°07'59" N & 75°05'20" W	*C. c. fuscus*
MT512608.1	MT512608_CcfCol35	Colombia	San Luis, Tolima	4°07'59" N & 75°05'20" W	*C. c. fuscus*
MT512589.1	MT512589_CcfCol35	Colombia	Ibague, Tolima	4°25'39" N & 75°08'03" W	*C. c. fuscus*
MT512590.1	MT512590_CcfCol35	Colombia	Ibague, Tolima	4°25'39" N & 75°08'03" W	*C. c. fuscus*
MT512591.1	MT512591_CcfCol35	Colombia	Ibague, Tolima	4°25'39" N & 75°08'03" W	*C. c. fuscus*
MT512592.1	MT512592_CcfCol35	Colombia	Ibague, Tolima	4°25'39" N & 75°08'03" W	*C. c. fuscus*
MT512593.1	MT512593_CcfCol35	Colombia	Ibague, Tolima	4°25'39" N & 75°08'03" W	*C. c. fuscus*
MT512605.1	MT512605_CcfCol35	Colombia	Prado, Tolima	3°42'49" N & 74°53'14" W	*C. c. fuscus*

MT512609.1	MT512609_CcfCol35	Colombia	San Luis, Tolima	4°07'59" N & 75°05'20" W	*C. c. fuscus*
MT512610.1	MT512610_CcfCol35	Colombia	San Luis, Tolima	4°07'59" N & 75°05'20" W	*C. c. fuscus*
MT512611.1	MT512611_CcfCol35	Colombia	San Luis, Tolima	4°07'59" N & 75°05'20" W	*C. c. fuscus*
MT512612.1	MT512612_CcfCol35	Colombia	San Luis, Tolima	4°07'59" N & 75°05'20" W	*C. c. fuscus*
MN130135	MN130135 *	Colombia	Isla Gorgona, Cauca	2°58'07" N & 78°11'03" W	*C. crocodilus*
MN130136	MN130136 *	Colombia	Isla Gorgona, Cauca	2°58'07" N & 78°11'03" W	*C. crocodilus*
MN130137	MN130137 *	Colombia	Isla Gorgona, Cauca	2°58'07" N & 78°11'03" W	*C. crocodilus*
MN130138	MN130138 *	Colombia	Isla Gorgona, Cauca	2°58'07" N & 78°11'03" W	*C. crocodilus*
MN130139	MN130139 *	Colombia	Isla Gorgona, Cauca	2°58'07" N & 78°11'03" W	*C. crocodilus*
MN218459	MN218459 *	Colombia	Isla Gorgona, Cauca	2°58'07" N & 78°11'03" W	*C. crocodilus*
MN218460	MN218460 *	Colombia	Isla Gorgona, Cauca	2°58'07" N & 78°11'03" W	*C. crocodilus*
MN218461	MN218461 *	Colombia	Isla Gorgona, Cauca	2°58'07" N & 78°11'03" W	*C. crocodilus*
MN218462	MN218462 *	Colombia	Isla Gorgona, Cauca	2°58'07" N & 78°11'03" W	*C. crocodilus*
MN218463	MN218463 *	Colombia	Isla Gorgona, Cauca	2°58'07" N & 78°11'03" W	*C. crocodilus*
MN218464	MN218464 *	Colombia	Isla Gorgona, Cauca	2°58'07" N & 78°11'03" W	*C. crocodilus*
MN218465	MN218465 *	Colombia	Isla Gorgona, Cauca	2°58'07" N & 78°11'03" W	*C. crocodilus*
MN218466	MN218466 *	Colombia	Isla Gorgona, Cauca	2°58'07" N & 78°11'03" W	*C. crocodilus*
MN218467	MN218467 *	Colombia	Isla Gorgona, Cauca	2°58'07" N & 78°11'03" W	*C. crocodilus*
MN218468	MN218468 *	Colombia	Isla Gorgona, Cauca	2°58'07" N & 78°11'03" W	*C. crocodilus*

* This study
